# Comparison of Clinical and Radiological Outcomes Between Different (Balloon) Guide Catheter With and Without Inflated Balloon in Acute Ischemic Stroke Patients: A MaSQ-Registry Study

**DOI:** 10.1007/s00270-024-03718-9

**Published:** 2024-04-19

**Authors:** R. R. M. M. Knapen, M. Celen, F. M. E. Pinckaers, B. A. J. M. Wagemans, W. H. van Zwam, R. J. van Oostenbrugge, C. van der Leij

**Affiliations:** 1 Department of Radiology and Nuclear Medicine, Maastricht University Medical Center+ and School for Cardiovascular Diseases (CARIM), Maastricht University, Maastricht, The Netherlands; 2 Department of Neurology, Zuyderland MC, Heerlen, Sittard-Geleen, The Netherlands; 3 Department of Radiology and Nuclear Medicine, Maastricht University Medical Center+, Maastricht, The Netherlands; 4 Department of Neurology, Maastricht University Medical Center+ and School for Cardiovascular Diseases (CARIM), Maastricht, The Netherlands; 5 Present Address: School for Cardiovascular Diseases Maastricht (CARIM), Maastricht University, Maastricht, The Netherlands

**Keywords:** Balloon guide catheter (BGC), Acute ischemic stroke (AIS), Endovascular treatment (EVT), Stroke

## Abstract

**Purpose:**

Balloon guide catheters (BGCs) are used in endovascular treatment (EVT) for ischemic stroke. Previous literature did not distinguish between BGC use with and without inflated balloon. This study aims to compare outcomes between non-BCG and BGC use with and without inflated balloon during EVT.

**Methods:**

Patients who underwent EVT for anterior circulation ischemic stroke between September 2020 and February 2023 were analyzed. Patients were divided into three groups: non-BGC, BGC with inflated balloon, or BGC without inflated balloon. The primary outcome was the ordinal modified Rankin Scale (mRS) at 90-day follow-up. Secondary outcomes included expanded Thrombolysis In Cerebral Ischemia score (eTICI) and periprocedural complications. Regression analyses with BGC with inflated balloon as comparator were performed with adjustments. Subgroup analyses were conducted based on first-line thrombectomy technique.

**Results:**

Out of 511 patients, 428 patients were included. Compared to BCG with inflated balloon, the mRS at 90 days did not differ in the group without inflated balloon (adjusted common [ac]OR: 1.07, 95%CI 0.67–1.73) or non-BGC (acOR: 1.42, 95%CI 0.83–2.42). Compared to patients treated with a BGC with inflated balloon, those treated with BGC without inflated balloon had lower eTICI scores (acOR: 0.59, 95%CI 0.37–0.94), and patients treated with non-BGC had lower chances of periprocedural complications (aOR: 0.41, 95%CI 0.20–0.86).

**Conclusions:**

This study shows no clinical differences in ischemic stroke patients treated with BGC with inflated balloon compared to non-BGC and BGC without inflated balloon, despite lower periprocedural complication rates in the non-BGC group and lower eTICI scores in the BGC without inflated balloon group.

**Level of Evidence:**

Level 3, non-controlled retrospective cohort study.

**Graphical Abstract:**

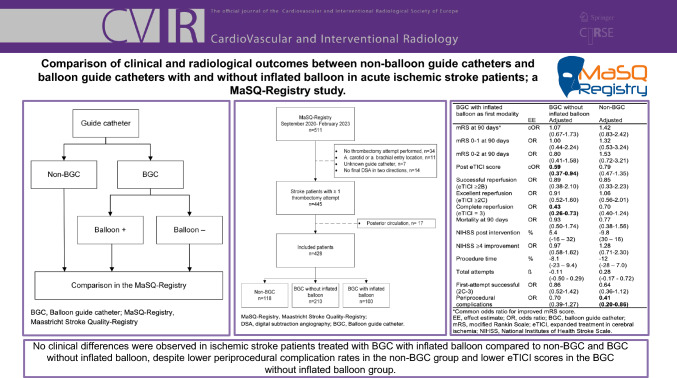

**Supplementary Information:**

The online version contains supplementary material available at 10.1007/s00270-024-03718-9.

## Introduction

Balloon guide catheters (BGCs) are often used during endovascular treatment (EVT) in patients with acute ischemic stroke (AIS) due to large vessel occlusion (LVO) in the anterior circulation. By inflating the balloon at the tip of the catheter during thrombectomy, antegrade flow arrest in the internal carotid artery is achieved, which is assumed to decrease fragmentation of the thrombus and subsequent embolization to new/other vascular territories [1–4]. When patients have a significant carotid stenosis, treating physicians may choose to not inflate the balloon since flow arrest is already achieved.

Most literature suggests that the use of BCGs during EVT improves clinical, technical, and safety outcomes when compared to non-balloon guide catheters (non-BGCs) [2, 5–11]. While some studies show equal or even worse technical outcomes when using a BGC [12, 13]. In these studies, however, no distinguishment is made between the use of a BGC with inflated or without inflated balloon. As it may be argued that the working mechanism of a BCG without inflated balloon is the same as a non-BGC, outcomes of the effectiveness of the BGC may therefore be overestimated.

The aim of the present study is to compare clinical and technical outcomes between BGC with and without inflated balloon and non-BGC in stroke patients registered within the Maastricht Stroke Quality-Registry (MaSQ-Registry).

## Methods

### Design and Participants

For this study, we used data from the Maastricht Stroke Quality-Registry (MaSQ-Registry) from September 2020 to February 2023. In the MaSQ-Registry, data were prospectively collected for quality purposes by the treating physicians and researchers. Patients were included if they met the following inclusion criteria: age ≥ 18 years, an intracranial anterior circulation occlusion (thrombus or dissection) confirmed by CT-angiography, and groin puncture within 24 h after symptom onset. Patients were excluded when no data were available regarding the guide catheter and the balloon, or when no final digital subtracted angiography was performed in two different projections. Patients were divided into three groups based on the first-line thrombectomy technique: non-BGC, BGC with inflated balloon, and BGC without inflated balloon.

Ethical approval for retrospective analysis was obtained from the medical ethics committee. The need to obtain individual informed consent was waived, according to the Dutch Medical Research Involving Human Subjects Act. Data were collected in a secured online Castor (v2023.1.2.0) database. This study was conducted using the STROBE guidelines.

### Outcome Measures

The primary outcome was the modified Rankin Scale 90 days after EVT. The mRS runs from 0 (no symptoms) to 6 (death) [14]. Secondary clinical outcomes included excellent and favorable functional outcome (defined as mRS 0–1 and mRS 0–2, respectively), the National Institutes of Health Stroke Scale (NIHSS) 24–48 h after the EVT, and early neurological recovery, defined as improvement of 4 or more points on the NIHSS at 24–48 h after EVT. Deceased patients were assigned a score of “42” [15]. The NIHSS was scored as standard care by the attending physician at the ward or retrospectively using a standardized score chart based on the reports of the neurological exam.

Technical outcomes were reperfusion rate, procedure duration, first-attempt successful reperfusion, and the occurrence of periprocedural complications. The expanded Thrombolysis In Cerebral Infarction (eTICI) scale was used to assess the reperfusion after EVT. eTICI is a scale from 0 (no perfusion) to 3 (100% reperfusion) [16]. Successful, excellent, and complete reperfusion was defined as eTICI ≥ 2B, ≥ 2C, and 3, respectively. The eTICI was scored by the treating physician at the end of the procedure.

### Imaging Assessment

The Alberta Stroke Program Early CT Score (ASPECTS) and the collateral status according to Tan et al. on baseline CT scans were assessed in a core laboratory by one neuroradiologist and one neuro-interventional radiologist [17]. Both core laboratory members were blinded for clinical outcome. After every thrombectomy attempt and at the end of the thrombectomy, the eTICI score was scored by the treating physician, who performed the thrombectomy. The final eTICI score was used as outcome measure and, when necessary, reevaluated by a second neuro-interventional radiologist.

### Treatment

Local and national guidelines for the treatment of AIS were followed. The choice of technique and devices during EVT was left to the treating physician. The EVT characteristics regarding the first-line technique, including the use of a (non-)BGC and whether the balloon was inflated or not, were registered by the treating physician directly after the procedure. There was no default guide catheter and no predefined criteria whether the balloon of a BGC should be inflated or not. These decisions were made by the treating physician based on personal preferences and periprocedural findings (e.g., when achieving flow arrest without inflating the balloon, when the BGC was not placeable in the internal carotid artery, in case of a carotid dissection). The reasons not to inflate the balloon were not registered. The first-line technique was registered as direct aspiration only, stent retriever thrombectomy only, or combined technique thrombectomy (stent retriever combined with an aspiration catheter). Regardless the used first-line technique, aspiration at the back end of the guide catheter was achieved using manual aspiration with a 50 cc syringe. Periprocedural complications included distal thrombi, vasospasm, perforations, and dissections and were scored by the treating physician directly after the EVT.

### Statistical Analysis

Baseline characteristics were analyzed with descriptive statistics. To analyze continuous variables, we used an ANOVA or Kruskal–Wallis test. For binary and ordinal data, the Chi-square test or Fisher’s exact test was used. Multivariable ordinal regression analysis was used to compare the differences in the mRS at 90 days with BGC with inflated balloon as comparator. Multivariable linear, ordinal, or binary logistic regression analyses were performed for our secondary outcomes as appropriate. Continuous outcome measures were transformed using the natural logarithm because the residuals were not normally distributed. Effect estimates from the resulting regression models were exponentiated to calculate the percentual change using the following formula: (exponentiate (*β*-coefficient) − 1) × 100%.

The adjustments used in the regression analyses were based on literature and univariable analyses. Variables used in the regression analyses for adjustments were age, atrial fibrillation, pre-NIHSS, pre-mRS (dichotomized 0 versus 1–5), systolic blood pressure, presence of a tandem lesion, time between symptom onset and groin puncture, baseline ASPECTS, and baseline collateral score. All analyses were performed using R (version 4.1.2). P values below 0.05 were considered statistically significant.

### Missing Values

Baseline characteristics were described using raw data. For the regression analyses, missing data were imputed with multiple imputation by chained equations (MICE) using the *mice* package version 3.14.0 with predefined variables as predictors. The number of imputations was set to 50. Table 1 shows the missing rate in baseline variables. The mRS-score was missing in 6.8%. When one of the subitems of the NIHSS or the total NIHSS score was missing (in 8.9% of the cases), the NIHSS sum score was imputed.

**Table 1:** The baseline characteristics of the included patients

	Non-BGC (*n* = 118)		BGC without inflated balloon (*n* = 210)		BGC with inflated balloon (*n* = 100)		*p* value	Missing (%)
Age—mean (SD)	74.7	(13)	73.4	(13)	72.9	(15)	0.57	0
Male sex—*n* (%)	56	(48)	93	(44)	50	(50)	0.62	0
NIHSS—median [IQR]	16	[10–20]	15	[9.0–20]	15	[10–21]	0.73	2.3
IVT given—*n* (%)	61	(52)	109	(52)	48	(48)	0.80	0
Systolic blood pressure—mean mmHg (SD)	165	(30)	161	(30)	161	(32)	0.43	4.7
Medical history—*n* (%)								
Pre-mRS—*n* (%)							0.81	35
0	40	(57)	76	(53)	33	(52)		
1	13	(19)	34	(24)	14	(22)		
2	10	(14)	19	(13)	12	(19)		
> 2	7	(9.9)	15	(10)	4	(6.4)		
Ischemic stroke	20	(17)	37	(18)	16	(16)	0.93	1.6
Atrial fibrillation	17	(15)	36	(18)	24	(24)	0.21	2.3
Hypertension	52	(44)	79	(39)	41	(41)	0.65	2.3
Hypercholesterolemia	13	(11)	30	(15)	13	(13)	0.67	2.1
Diabetes Mellitus	12	(10)	29	(14)	13	(13)	0.63	2.1
Current smoking	19	(28)	44	(30)	16	(24)	0.64	34
Usage of Coumarine	8	(7.1)	12	(5.9)	8	(8.3)	0.75	4.0
Usage of Anticoagulation	13	(11)	23	(11)	16	(16)	0.43	3.0
Usage of Antiplatelet	35	(30)	69	(33)	34	(35)	0.76	0.9
Imaging								
Collaterals—*n* (%)							0.49	6.8
Grade 0	0	(0)	4	(2.0)	2	(2.2)		
Grade 1	45	(43)	92	(46)	42	(46)		
Grade 2	55	(52)	98	(49)	47	(51)		
Grade 3	6	(5.7)	7	(3.5)	1	(1.1)		
ASPECTS—median [IQR]	9	[7–10]	9	[7–10]	8	[7, 8]	0.08	6.3
Occlusion location on CTA—*n* (%)							0.71	0
ICA	6	(5.1)	6	(2.9)	4	(4.0)		
ICA-T	14	(12)	28	(13)	17	(17)		
MCA segment M1	60	(51)	124	(59)	54	(54)		
MCA segment M2	37	(31)	51	(24)	25	(25)		
ACA segment A1	1	(0.8)	1	(0.5)	0	(0)		
Tandem lesion—n (%)							0.002	0
No stenosis (< 50%)	88	(75)	145	(69)	89	(89)		
Stenosis (50–99%)	17	(14)	34	(16)	8	(8)		
Occlusion (100%)	13	(11)	13	(15)	3	(3)		
Workflow								
Transfer from primary stroke center—*n* (%)	92	(78)	162	(77)	70	(70)	0.31	0
Onset to groin—median minutes [IQR]	197	[147–393]	213	[152–396]	202	[150–359]	0.97	3.0
First-line technique—*n* (%)							0.10	0.2
Combined	47	(40)	83	(40)	54	(54)		
Aspiration only	71	(60)	125	(60)	45	(45)		
Stent retriever only	0	(0)	1	(0.5)	1	(1.0)		

*BGC* balloon guide catheter, *SD* standard deviation, *NIHSS* National Institutes of Health Stroke Scale, *IVT* intravenous thrombolysis, *IQR* interquartile range, *mRS* modified Rankin Scale, *ASPECTS* Alberta Stroke Program Early CT Score, *CTA* CT-angiography, *ICA* internal carotid artery, *ICA-T* internal carotid artery terminus, *MCA* middle cerebral artery, *M1* horizontal segment of the middle cerebral artery, *M2* insular segment of the middle cerebral artery, *ACA* anterior cerebral artery, *A1* first segment of the anterior cerebral artery

### Subgroup Analyses

To investigate the effect of the first-line technique on the mRS score, subgroup analyses were performed. We distinguished direct aspiration only thrombectomy, stent retriever only thrombectomy, and the combined technique thrombectomy as first-line thrombectomy techniques. In the subgroup analyses, the same adjustments were made as in the main analyses. Additionally, a sensitivity analysis was performed to investigate the effect of the different guide catheters after removing the patients with a carotid artery stenosis or a carotid stent placement during EVT. The analysis was repeated on the mRS at 90 days, mRS 0–1, and mRS 0–2.

## Results

### Baseline Characteristics

**Fig. 1:**
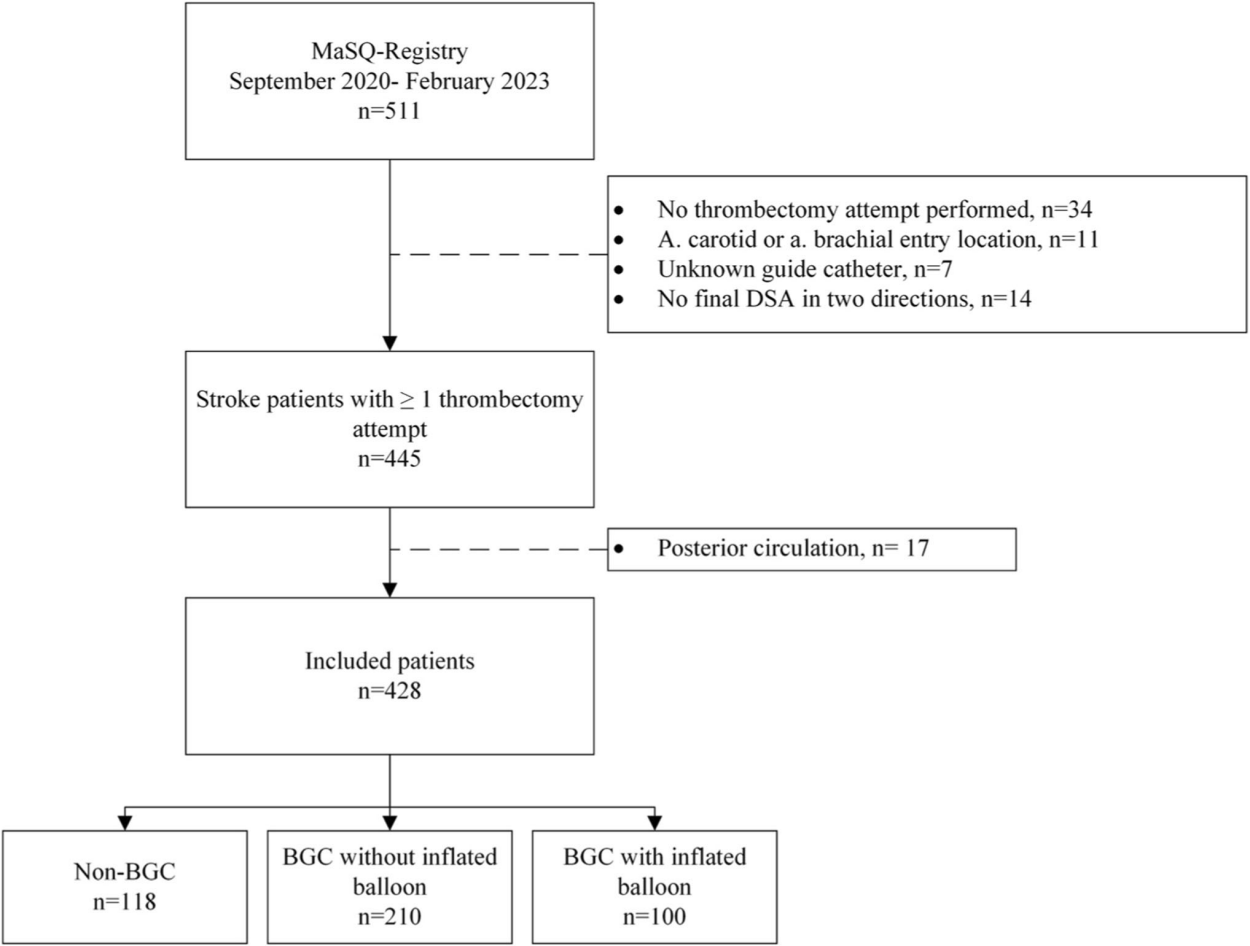
Flowchart of included patients. MaSQ-Registry, Maastricht Stroke Quality-Registry; DSA, digital subtraction angiography; BGC, Balloon guide catheter

Out of 511 eligible patients in the MaSQ-Registry, a total of 428 patients were included (Fig. 1). A total of 310 patients were treated with a BGC (72%) of which 210 patients (68%) were treated with a BGC without inflated balloon. Table S1 overviews all used guide catheters. A 9Fr BGC was most often used in patients treated with BGC with inflated balloon (97%) and without inflated balloon (99%), while an 8Fr or smaller long sheath was most often used in the non-BGC group (96%). Baseline characteristics are presented in Table 1. The ASPECT score differed between the groups (P = 0.04). An ASPECT score of 8–10 was mostly seen in the non-BGC group (75%), followed by BGC without inflated balloon group (69%) and BGC with inflated balloon group (57%). No other significant differences in baseline characteristics were found.

### Clinical Outcome

**Fig. 2:**
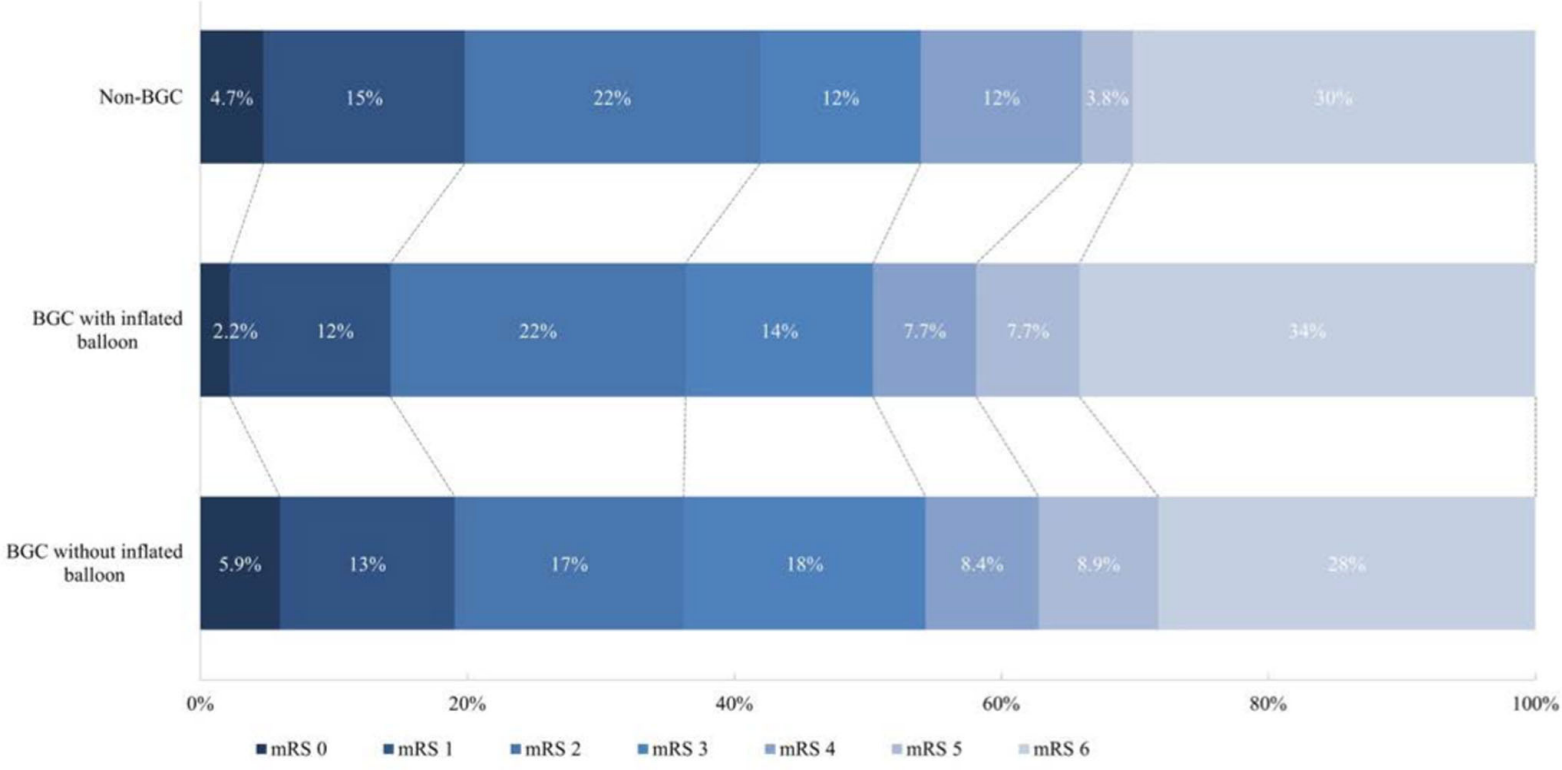
Distribution of modified Rankin Scale score at 90 days follow-up between non-BGC, BGC with inflated balloon, and BGC without inflated balloon. Multiple ordinal regression with BGC with inflated balloon as comparator showed no differences compared to BGC without inflated balloon (acOR: 1.07, 95%CI 0.67–1.73) and non-BGC (acOR: 1.42, 95%CI 0.83–2.42). BGC, Balloon guide catheter; mRS, modified Rankin scale

The clinical outcomes are described in Table 2. Results of the regression analyses are presented in Table 3. Compared to the group with BCG with inflated balloon, the mRS score at 90 days post-EVT did not differ significantly in the non-BGC (adjusted common [ac]OR: 1.42, 95%CI 0.83–2.42) or the BGC without inflated balloon group (acOR: 1.07, 95%CI 0.67–1.73) (Fig. 2). Favorable outcome (mRS 0–2) at 90 days and NIHSS improvement of ≥ 4 did not differ significantly (Table 3).

**Table 2:** Outcome measures between the three groups

	Non-BGC (*n* = 118)		BGC without inflated balloon (*n* = 210)		BGC with inflated balloon (*n* = 100)	
mRS at 90 days^#^—*n* (%)						
0	5	(4.7)	12	(5.9)	2	(2.2)
1	16	(15)	26	(13)	11	(12)
2	23	(22)	35	(17)	20	(22)
3	13	(12)	37	(18)	13	(14)
4	13	(12)	17	(8.4)	7	(7.7)
5	4	(3.8)	18	(8.9)	7	(7.7)
6	32	(30)	57	(28)	31	(34)
mRS score 0–1^#^—*n* (%)	21	(20)	38	(19)	13	(14)
mRS score 0–2^#^—*n* (%)	44	(42)	73	(36)	33	(36)
Successful reperfusion (eTICI ≥ 2B)—*n* (%)	105	(89)	188	(90)	91	(91)
Excellent reperfusion (eTICI ≥ 2C)—*n* (%)	87	(74)	149	(71)	73	(73)
Complete reperfusion (eTICI = 3)—*n* (%)	43	(36)	55	(26)	46	(46)
Mortality at 90 days—*n* (%)	32	(30)	57	(28)	31	(34)
NIHSS post intervention^^^—median [IQR]	7	[2.0–17]	9	[4.0–16]	10	[2.5–17]
NIHSS ≥ 4 improvement^^^—*n* (%)	60	(56)	94	(50)	44	(51)
Procedure time –median minutes [IQR]	26.5	[15–45]	30.0	[16–45]	25.0	[15–46]
Total attempts—median [IQR]	2	[1–3]	1	[1–3]	1	[1–3]
First-attempt successful (2C-3)—*n* (%)	41	(35)	89	(42)	47	(47)
Periprocedural complications—*n* (%)	16	(14)	45	(21)	25	(25)

^#^mRS was missing in 29 patients; ^ NIHSS was missing in 38 patients

*BGC* balloon guide catheter, *mRS* modified Rankin scale, *eTICI* expanded treatment in cerebral ischemia, *NIHSS* National Institutes of Health Stroke Scale, *IQR* interquartile range

**Table 3:** Associations between clinical and technical outcomes and the use of a (non-)BGC

BGC with inflated balloon as first modality	EE	BGC without inflated balloon		Non-BGC	
		Unadjusted	Adjusted	Unadjusted	Adjusted
mRS at 90 days*	cOR	1.25 (0.82–1.92)	1.07 (0.67–1.73)	1.40 (0.86–2.27)	1.42 (0.83–2.42)
mRS 0–1 at 90 days	OR	1.32 (0.68–2.58)	1.00 (0.44–2.24)	1.53 (0.73–3.21)	1.32 (0.53–3.24)
mRS 0–2 at 90 days	OR	1.06 (0.64–1.76)	0.80 (0.41–1.58)	1.41 (0.81–2.47)	1.53 (0.72–3.21)
Post eTICI score	cOR	**0.57** **(0.36–0.90)**	**0.59** **(0.37–0.94)**	0.77 (0.46–1.28)	0.79 (0.47–1.35)
Successful reperfusion (eTICI ≥ 2B)	OR	0.85 (0.37–1.91)	0.89 (0.38–2.10)	0.80 (0.33–1.96)	0.85 (0.33–2.23)
Excellent reperfusion (eTICI ≥ 2C)	OR	0.88 (0.52–1.50)	0.91 (0.52–1.60)	0.91 (0.52–1.60)	1.06 (0.56–2.01)
Complete reperfusion (eTICI = 3)	OR	**0.41** **(0.25–0.67)**	**0.43** **(0.26–0.73)**	0.67 (0.39–1.16)	0.70 (0.40–1.24)
Mortality at 90 days	OR	0.74 (0.44–1.25)	0.93 (0.50–1.74)	0.78 (0.44–1.40)	0.77 (0.38–1.56)
NIHSS post intervention	%	0.6 (− 22–30)	5.4 (− 16–32)	− 11.8 (− 34–17)	− 9.8 (30–16)
NIHSS ≥ 4 improvement	OR	0.95 (0.58–1.55)	0.97 (0.58–1.62)	1.22 (0.70–2.14)	1.28 (0.71–2.30)
Procedure time	%	− 2.7 (− 18–16)	− 8.1 (− 23–9.4)	− 6.0 (− 23–14)	− 12 (− 28–7.0)
Total attempts	ß	− 0.15 (− 0.54–0.24)	− 0.11 (− 0.50–0.29)	0.24 (− 0.19 − 0.67)	0.28 (− 0.17–0.72)
First-attempt successful (2C-3)	OR	0.81 (0.50–1.31)	0.86 (0.52–1.42)	0.60 (0.35–1.04)	0.64 (0.36–1.12)
Periprocedural complications	OR	0.82 (0.47–1.43)	0.70 (0.39–1.27)	**0.47** **(0.23–0.94)**	**0.41** **(0.20–0.86)**

*Common odds ratio for improved mRS score

*EE* effect estimate, *cOR* common odds ratio, *OR* odds ratio, *BGC* balloon guide catheter, *mRS* modified Rankin scale, *eTICI* expanded treatment in cerebral ischemia, *NIHSS* National Institutes of Health Stroke Scale

### Technical Outcome

Successful reperfusion rates (eTICI ≥ 2B) were comparable between the non-BGC, BGC with inflated balloon, and BGC without inflated balloon (89 90, and 91%, respectively; Table 2). In the patient group treated with BGC without inflated balloon, significantly lower complete reperfusion (eTICI 3) rates were observed compared to patients treated with BGC with inflated balloon (26 vs. 46%; aOR: 0.43, 95%CI 0.26–0.73) (Table 3).

Additionally, the ordinal eTICI score was also lower in patients treated with BGC without inflated balloon compared to the BGC with inflated balloon group (acOR: 0.59, 95%CI 0.37–0.94). Procedure time did not differ between the groups (Tables 2, 3).

Periprocedural complications were lowest in the non-BGC group (14%), followed by the BGC without inflated balloon group (21%), and BGC with inflated balloon group (25%) (Table 2). Regression analyses showed lower odds of complications in the non-BGC group compared to the BGC with inflated balloon group (aOR: 0.41, 95%CI 0.20–0.86) (Table 3). Table S2 gives an overview of all complications between the three groups.

### Subgroup Analyses

Two patients were treated with stent retriever as first-line thrombectomy technique without direct aspiration; therefore, these patients were not taken into account in the subgroup analyses. Patients treated with combined aspiration and stent retriever thrombectomy as first-line technique did not differ on clinical and technical outcomes when using a non-BGC, BGC with inflated balloon, or BGC without inflated balloon (Tables S3, S4).

When patients were treated with direct aspiration thrombectomy as first-line technique, patients treated with a non-BGC had higher chances for a better clinical outcome (less disability) at 90 days compared to the BGC with inflated balloon group (acOR: 2.79, 95%CI 1.30–5.95), while no differences were seen between the BGC without inflated balloon group compared to BGC with inflated balloon group (acOR: 1.50, 95%CI 0.75–2.97) (Tables S5, S6). The mortality rate at 90 days and the periprocedural complications rate were lower in patients treated with non-BGC compared to BGC with inflated balloon (aOR: 0.33, 95%CI 0.12–0.91 and aOR: 0.35, 95%CI 0.13–0.996, respectively), whereas the mortality and periprocedural complications rate were comparable between the BGC without inflated balloon group and BGC with inflated balloon group (aOR: 0.72, 95%CI 0.30–1.71 and aOR: 0.67, 95%CI 0.29–1.55, respectively). Regression analyses did not show differences in complete reperfusion rates (Table S6).

Sixteen patients with a carotid artery stenosis and eighteen patients with a stent placement during EVT were excluded for the sensitivity analysis. The mRS at 90 days, mRS 0–1, and mRS 0–2 did not differ between the groups (Table S7).

## Discussion

In this observational MaSQ-Registry study, we compared clinical and technical outcomes between the use of BGC with or without inflated balloon and non-BGC during EVT in AIS patients with anterior LVO. No differences were seen in clinical outcomes between the groups. Non-BGC showed lower chances of periprocedural complications compared to BGC with inflated balloon, whereas the BGC without inflated balloon group had lower eTICI scores compared to the BGC with inflated balloon group. Subgroup analyses showed higher odds of a shift toward better mRS score at 90 days, lower mortality rates, and less periprocedural complications rates when a non-BGC was used with direct aspiration thrombectomy only, compared to BGC with inflated balloon. No differences in subgroup analyses were seen between the BGC with and without inflated balloon.

To the best of our knowledge, previous literature on the effect of inflating the balloon on outcomes is not available. It is remarkable that, in our study, the balloon was not inflated in 68% of the cases in which a BGC was used. The choice (not) to inflate the balloon of the BGC was left to the treating physicians. An explanation to withhold inflating the balloon may be personal preference, the occurrence of a dissection, and when flow arrest was already achieved due to a stenosis or occlusion in the carotid artery. Although numbers regarding flow arrest were not registered in the MaSQ-Registry properly, our results partly substantiate this last theory, as 22% of patients treated with a BGC without inflated balloon had a carotid stenosis or occlusion, whereas this percentage was 11% in patients in which a BGC with inflated balloon was used. Since our sensitivity analysis showed comparable clinical outcomes between a non-BGC and a BGC without inflated balloon, one may consider opting for a (cheaper) non-BGC when a stenosis or occlusion is seen on the pre-EVT CT-angiography. Another explanation for the low rate of inflated balloons may be the placement of the BGC in the internal carotid artery. It is known that distal placement of the BGC in the internal carotid artery has higher rates of achieving first-attempt successful recanalization compared to a more proximally placed BGC [18]. When treating physicians have difficulties in placing the BGC, they may choose to not inflate the balloon since they estimate the risks of complications higher.

Previously published studies have reported higher rates of successful reperfusion (eTICI ≥ 2B) when using a BGC in combination with a stent retriever (84%-94%) compared to a non-BGC (75–76%) [6, 7]. We could not confirm these results. Notably, regardless of (balloon) guide catheter use, rates of successful reperfusion observed in this daily practice registry were comparable to previous literature [6, 7].

The overall procedural complication rate was lower when using a non-BGC compared to a BGC with inflated balloon (aOR: 0.41, 95%CI 0.20–0.86). This result is in contrast to the literature, as previous studies have reported lower or comparable rates of complications when using a BGC with inflated balloon compared to a non-BGC [19, 20]. This difference in complication rate may be partly explained by the way complications were registered in the current study. In our registry, procedural complications were registered directly after the EVT by the threating physician and not by a full core laboratory, which might have detected more complications such as distal emboli. This might lead to an underestimation of some procedural complications and an overestimation of the reperfusion rates.

It is known that first-line technique might have an additional influence on the clinical outcomes [21]. The use of a stent retriever only in combination with a BGC improves outcomes compared to stent retriever in combination with a non-BGC [5–8, 10, 11]. In our study, only two patients were treated with stent retriever only as first-line technique. This indicates that in our center, this technique has largely been replaced by the combined technique. With the increased use of the combined first-line technique, the added value of a BGC may have become smaller.

Regarding the first-line thrombectomy technique, a meta-analysis showed improved clinical and procedural outcomes when using a BGC compared to a non-BGC when stent retriever only or direct aspiration only was used as first-line technique, but not when a combined technique was used as first-line technique [21]. This is in line with some literature showing no effect of using a BGC versus a non-BGC when the combined technique was used [12, 20]. In subgroup analyses, we observed comparable results between the BGC groups when the combined technique was used as first-line technique. This potentially means that the added value of a BGC is less in the combined technique as first-line compared to stent retriever only thrombectomy.

When looking at direct aspiration thrombectomy only, better mRS scores (less disability), lower mortality, and complications rates were observed when direct aspiration was combined with non-BGC compared to a BGC with inflated balloon, despite lower successful reperfusion rates (non-BGC: 89% and BGC with inflated balloon: 98%). These results contradict the aforementioned meta-analysis [21]. However, our subgroup analyses need to be interpreted with caution, as they were performed with the same adjustments as in the main analysis.

## Limitations

This study has several limitations. First, this was a single-center study, limiting external validity. Second, the use of (non-)BGC, the choice to inflate the balloon, and the duration of the inflation during EVT were left to the treating physician, potentially introducing selection bias. Third, the NIHSS before and after EVT was partly scored retrospectively if insufficient data were available from the attending physician. Fourth, no numbers were available regarding patients with a dissection at baseline. Since placing and inflating a BGC in these patients could be challenging, this may introduce potential bias. On the other hand, this study was conducted in a tertiary comprehensive stroke center with experienced treating physicians. Second, our data represent real-world daily practice, which may make our results better generalizable to other centers. Third, to our knowledge, limited data are known on comparing differences in outcomes of EVT in patients treated using a BGC with and without inflated balloon. The ProFATE Trial is a randomized controlled trial investigating the use of a BGC with inflated balloon versus a BGC without inflated balloon [22]. Results are expected in 2024 and will hopefully give further insights.

## Conclusions

This single-center study shows no differences between non-BGC and BGC with or without inflated balloon regarding clinical outcomes in ischemic stroke patients due to anterior LVO treated with EVT. Compared to the BGC with inflated balloon group, lower procedural complication rates were observed in the non-BGC group and lower eTICI score in the BGC without inflated balloon group.

### Supplementary Information

Below is the link to the electronic supplementary material.

Supplementary file1 (PDF 116 kb)
