# Supplementary material for: Comparison of Clinical and Radiological Outcomes Between Different (Balloon) Guide Catheter With and Without Inflated Balloon in Acute Ischemic Stroke Patients: A MaSQ-Registry Study
Source: Cardiovasc Intervent Radiol. 2024 Apr 19;47(7):918–28. doi: 10.1007/s00270-024-03718-9 (PMC11239717; doi:10.1007/s00270-024-03718-9)
Supplement: Supplementary file 1 — Supplementary file1 (PDF 116 kb) [file 270_2024_3718_MOESM1_ESM.pdf]

Supplemental

Table S1. Overview of used guide catheters

	n/N	(%)
Non-BGC		
Arrowsheath 6 Fr – n. (%)	1/118	0.8
Arrowsheath 8Fr – n. (%)	72/118	61
Arrowsheath 9Fr – n. (%)	5/118	4.2
Cerebase 8Fr – n. (%)	38/118	32
Durasheath 8 Fr – n. (%)	1/118	0.8
Envoy 6 Fr – n. (%)	1/118	0.8
BGC without inflated balloon		
Meri balloon guide catheter 8Fr – n. (%)	1/210	0.5
Meri balloon guide 9Fr – n. (%)	207/210	99
FlowGate balloon guide 8Fr – n. (%)	2/210	1.0
BGC with inflated balloon		
Meri balloon guide catheter 8Fr – n. (%)	2/100	2.0
Meri balloon guide 9Fr – n. (%)	94/100	94
FlowGate balloon guide 8Fr – n. (%)	1/100	1.0
FlowGate balloon guide 9Fr – n. (%)	3/100	3.0

BGC, balloon guide catheter; Fr, French.

Table S2. Overview of complications between the three groups

	Non-BGC (n=118)*		BGC without inflated balloon (n=210)#		BGC with inflated balloon (n=100)^	
Distal thrombus – n. (%)	3	2.5	15	7.1	11	11
Thrombus in new territory – n. (%)	2	1.7	8	3.8	3	3.0
Dissection – n. (%)	7	5.9	10	4.8	7	7.0
Perforation – n. (%)	2	1.7	4	1.9	5	5.0
Vasospasm – n. (%)	1	0.8	0	0	2	2.0
Other – n. (%)	3	2.5	9	4.3	2	2.0

*: in 3 patients 2 complications occurred; #: in 1 patient 2 complications occurred; ^: in 5 patients 2 complications occurred.

BGC, balloon guide catheter.

Table S3. Outcome measures between the three groups when combined thrombectomy technique was used as first-line thrombectomy technique.

	Non-BGC (n=47)		BGC without inflated balloon (n=83)		BGC with inflated balloon (n=54)	
mRS at 90 days# – n. (%)						
0	1	(2.3)	4	(5.0)	1	(2.0)
1	5	(12)	11	(14)	7	(14)
2	10	(23)	13	(16)	11	(22)
3	2	(4.7)	16	(20)	10	(20)
4	7	(16)	7	(8.8)	2	(4.0)
5	1	(2.3)	10	(13)	3	(6.0)
6	17	(40)	19	(24)	16	(32)
mRS score 0-1# – n. (%)	6	(14)	15	(19)	6	(16)
mRS score 0-2# – n.(%)	16	(37)	28	(35)	19	(38)
Successful reperfusion (eTICI ≥2B) – n. (%)	42	(89)	70	(84)	46	(85)
Excellent reperfusion (eTICI ≥2C) – n. (%)	33	(70)	52	(63)	37	(69)
Complete reperfusion (eTICI = 3) – n. (%)	16	(34)	21	(25)	23	(43)
Mortality at 90 days – n. (%)	17	(40)	19	(24)	16	(32)
NIHSS ≥ 4 improvement^ – n. (%)	20	(47)	37	(48)	21	(47)
Procedure time – median minutes [IQR]	31	[20-47]	36	[22-52]	29	[17-45]
Total attempts – median [IQR]	1	[1-3]	1	[1-2]	1	[1-3]
First-attempt successful (2C-3) – n. (%)	21	(45)	32	(39)	27	(50)

Per procedural complications – n. (%)	7	(15)	19	(23)	13	(24)

mRS was missing in 11 patients; ^ NIHSS was missing in 15 patients.

BGC, balloon guide catheter; mRS, modified Rankin Scale; eTICI, expanded treatment in cerebral ischemia; NIHSS, National Institutes of Health Stroke Scale.

Table S4. Associations between clinical and technical outcomes and the use of a (non-)BGC when combined thrombectomy technique was used as first-line thrombectomy technique.

BGC with inflated balloon as first modality	EE	BGC without inflated balloon Unadjusted	Adjusted	Non-BGC Unadjusted	Adjusted
mRS at 90 days*	cOR	1.12 (0.62-2.07)	0.89 (0.43-1.82)	0.81 (0.39-1.65)	0.69 (0.29-1.62)
mRS 0-1 at 90 days	OR	1.25 (0.50-3.11)	0.93 (0.29-2.96)	0.92 (0.30-2.82)	0.47 (0.11-2.04)
mRS 0-2 at 90 days	OR	0.93 (0.45-1.91)	0.74 (0.27-2.02)	1.04 (0.45-2.37)	1.06 (0.32-3.49)
Successful reperfusion (eTICI ≥2B)	OR	0.94 (0.36-2.45)	1.01 (0.33-3.09)	1.46 (0.44-4.86)	2.30 (0.55-9.60)
Excellent reperfusion (eTICI ≥2C)	OR	0.77 (0.37-1.60)	0.74 (0.32-1.70)	1.08 (0.46-2.55)	1.23 (0.47-3.23)
Complete reperfusion (eTICI = 3)	OR	**0.46 (0.22-0.95)**	0.47 (0.20-1.07)	0.70 (0.31-1.57)	0.67 (0.27-1.68)
Mortality at 90 days	OR	0.70 (0.32-1.52)	0.90 (0.32-2.56)	1.39 (0.60-3.20)	1.57 (0.50-4.90)
NIHSS ≥4 improvement	OR	0.97 (0.47-1.97)	1.12 (0.50-2.52)	0.90 (0.40-2.04)	1.10 (0.44-2.77)
Procedure time	%	7.5 (-15 - 36)	0.6 (-22 - 29)	2.5 (-22 - 34)	-7.6 (-31 - 22)
Total attempts	ß	-0.13 (-0.58 – 0.33)	-0.10 (-0.59 – 0.40)	-0.10 (-0.63 – 0.42)	-0.22 (-0.78 – 0.34)
First-attempt successful (2C-3)	OR	0.63 (0.31-1.26)	0.57 (0.26-1.23)	0.81 (0.37-1.78)	0.88 (0.37-2.09)
Per procedural complications	OR	0.94 (0.42-2.11)	0.71 (0.28-1.77)	0.55 (0.20-1.54)	0.46 (0.15-1.40)

*Common odds ratio for improved mRS score.

BGC, balloon guide catheter; mRS, modified Rankin Scale; eTICI, expanded treatment in cerebral ischemia; NIHSS, National Institutes of Health Stroke Scale.

Table S5. Outcome measures between the three groups when direct aspiration thrombectomy technique was used as first-line thrombectomy technique.

	Non-BGC (n=71)		BGC without inflated balloon (n=125)		BGC with inflated balloon (n=45)	
mRS at 90 days[#] – n. (%)						
0	4	(6.4)	8	(6.7)	1	(2.5)
1	11	(18)	15	(13)	4	(10)
2	13	(21)	22	(18)	8	(20)
3	11	(18)	19	(16)	3	(7.5)
4	6	(9.5)	10	(8.3)	5	(13)
5	3	(4.8)	8	(6.7)	4	(10)
6	15	(24)	38	(32)	15	(38)
mRS score 0-1[#] – n. (%)	15	(24)	23	(19)	5	(13)
mRS score 0-2[#] – n.(%)	28	(44)	45	(38)	13	(33)
Successful reperfusion (eTICI ≥2B) – n. (%)	63	(89)	116	(93)	44	(98)
Excellent reperfusion (eTICI ≥2C) – n. (%)	54	(76)	95	(76)	35	(78)
Complete reperfusion (eTICI = 3) – n. (%)	27	(38)	33	(26)	22	(49)
Mortality at 90 days – n. (%)	15	(24)	38	(32)	15	(38)
NIHSS ≥ 4 improvement[^] – n. (%)	40	(63)	56	(51)	22	(54)
Procedure time – median minutes [IQR]	22	[14-44]	23	[14-38]	23	[13-45]
Total attempts – median [IQR]	2	[1-4]	2	[1-3]	2	[1-4]
First-attempt successful (2C-3) – n. (%)	20	(28)	56	(45)	20	(44)
Periprocedural complications – n. (%)	9	(13)	26	(21)	12	(27)

mRS was missing in 18 patients; ^ NIHSS was missing in 23 patients.

BGC, balloon guide catheter; mRS, modified Rankin Scale; eTICI, expanded treatment in cerebral ischemia; NIHSS, National Institutes of Health Stroke Scale.

Table S6. Associations between clinical and technical outcomes and the use of a (non-)BGC when direct aspiration technique was used as first-line thrombectomy technique.

BGC with inflated balloon as first modality		BGC without inflated balloon		Non-BGC	
	EE	Unadjusted	Adjusted	Unadjusted	Adjusted
mRS at 90 days*	cOR	1.52 (0.81-2.86)	1.50 (0.75-2.97)	**2.22 (1.11-4.44)**	**2.79 (1.30-5.95)**
mRS 0-1 at 90 days	OR	1.49 (0.54-4.17)	1.31 (0.36-4.75)	2.18 (0.74-6.45)	3.13 (0.79-12.44)
mRS 0-2 at 90 days	OR	1.34 (0.63-2.84)	1.16 (0.41-3.27)	1.95 (0.87-4.39)	2.40 (0.79-7.28)
Successful reperfusion (eTICI ≥2B)	OR	0.29 (0.04-2.41)	0.16 (0.02-1.46)	0.18 (0.02-1.50)	0.37 (0.04-3.24)
Excellent reperfusion (eTICI ≥2C)	OR	0.87 (0.38-1.96)	1.01 (0.42-2.45)	0.91 (0.37-2.22)	1.04 (0.39-2.78)
Complete reperfusion (eTICI = 3)	OR	0.36 (0.18-0.73)	0.39 (0.19-0.82)	0.64 (0.30-1.37)	0.70 (0.31-1.56)
Mortality at 90 days	OR	0.71 (0.34-1.47)	0.72 (0.30-1.71)	0.46 (0.20-1.08)	**0.33 (0.12-0.91)**
NIHSS ≥4 improvement	OR	0.93 (0.45-1.88)	0.92 (0.44-1.93)	1.50 (0.68-3.30)	1.50 (0.65-3.45)
Procedure time	%	-3.9 (-25 - 24)	-8.7 (-29 - 18)	-5.4 (-28 - 25)	-8.9 (-31 - 21)
Total attempts	ß	-0.26 (-0.88 - 0.36)	-0.25 (-0.87 – 3.73)	0.36 (-0.32 – 1.03)	0.41 (-0.28 – 1.10)
First-attempt successful (2C-3)	OR	0.98 (0.49-1.96)	1.16 (0.56-2.40)	0.49 (0.22-1.08)	0.56 (0.24-1.28)
Per procedural complications	OR	0.72 (0.33-1.60)	0.67 (0.29-1.55)	0.40 (0.15-1.05)	**0.35 (0.13-0.996)**

*Common odds ratio for improved mRS score.

BGC, balloon guide catheter; mRS, modified Rankin Scale; eTICI, expanded treatment in cerebral ischemia; NIHSS, National Institutes of Health Stroke Scale.

Table S7. Sensitivity analyses on clinical outcomes between the use of a (non-)BGC when no carotid artery stenosis or carotid stenting was included.

BGC with inflated balloon as first modality		BGC without inflated balloon		Non-BGC	
	EE	Unadjusted	Adjusted	Unadjusted	Adjusted
mRS at 90 days*	cOR	1.05 (0.66-1.67)	1.01 (0.60-1.67)	1.19 (0.70-2.02)	1.27 (0.72-2.66)
mRS 0-1 at 90 days	OR	1.05 (0.51-2.18)	0.96 (0.39-2.36)	1.23 (0.54-2.80)	1.16 (0.42-3.19)
mRS 0-2 at 90 days	OR	0.88 (0.51-1.51)	0.66 (0.32-1.36)	1.22 (0.67-2.21)	1.32 (0.59-2.92)

*Common odds ratio for improved mRS score.

BGC, balloon guide catheter; mRS, modified Rankin Scale.
